# Reduced Brainstem Volume is Associated with Mobility Impairments in Youth with Cerebral Palsy

**DOI:** 10.21203/rs.3.rs-2566073/v1

**Published:** 2023-02-16

**Authors:** Michael P. Trevarrow, S. Shekar Dukkipati, Sarah E. Baker, Tony W. Wilson, Max J. Kurz

**Affiliations:** Boys Town National Research Hospital; Boys Town National Research Hospital; Boys Town National Research Hospital; Boys Town National Research Hospital; Boys Town National Research Hospital

**Keywords:** Cerebral Palsy, Lower Extremity, Gait, MRI

## Abstract

Persons with cerebral palsy (CP) have impaired mobility that has been attributed to changes in structure and function within the nervous system. The brainstem is a region that plays a critical role in locomotion by connecting the cortex and cerebellum to the spinal cord, yet this region has been largely unstudied in persons with CP. The objective of this investigation was to use high-resolution structural MRI and biomechanical analyses to examine whether the volume of the whole brainstem and its constituent elements are altered in CP, and if these alterations relate to the mobility impairments within this population. We assessed the volume of the pons, midbrain, medulla, and superior cerebellar peduncle (SCP) in a cohort of persons with CP (N = 26; Age = 16.3 ± 1.0 yrs; GMFCS levels I-IV, Females = 12) and a cohort of neurotypical (NT) controls (N = 38; Age = 14.3 ± 0.4 yrs, Females = 14) using structural MR imaging of the brainstem. Outside the scanner, a digital mat was used to quantify the spatiotemporal gait biomechanics of these individuals. Our MRI results revealed that there was a significant decrease in volume of the total brainstem, midbrain, and pons in persons with CP in comparison to the NT controls. Furthermore, we found that the altered volumes were related to reduced gait velocity and step length. These results suggest that there are structural changes in the brainstems of persons with CP that may contribute to the mobility impairments that are ubiquitous within this population.

## Introduction

Cerebral palsy (CP) is the most prevalent pediatric neurological disorder diagnosed in the United States and results in lifelong mobility impairments affecting daily function and overall quality of life [1–3]. These mobility impairments partially stem from musculoskeletal abnormalities such as spasticity, hyperexcitable reflexes, joint contractures, and strength deficits [4–7]. A growing body of literature now suggests that the musculoskeletal abnormalities may be driven by alterations in sensorimotor cortical activity [8–11]. However, it has also become increasingly evident that the insult to the developing brain experienced by persons with CP results in structural changes throughout the CNS that also play a major role in the detriments to overall motor function and mobility.

Structural MRI has effectively illustrated widespread anatomical alterations within both the brain and spinal cord in persons with CP. Specifically, diffusion tensor imaging (DTI) has revealed that damage to the thalamocortical tracts is associated with greater impairment to gross motor function and sensory processing [12–14]. Furthermore, structural changes have been noted across a wide range of cortical areas in persons with CP, including altered gray matter thickness within sensorimotor, occipital, temporal, parietal, and insular areas [15–17]. The decreased cortical thickness within the somatosensory cortices in turn results in reduced hand motor performance [16] and somatosensory cortical activity [18]. Similarly, decreases in both gray and white matter in the cervical-thoracic spinal cord has been shown in adults with CP, and these changes were directly connected with reduced hand dexterity [19]. Reductions in gray matter may be indicative of decreased cell size, cell number, and reduced dendritic arborization and synaptic density, while reductions in white matter may indicate changes in myelination that adversely affects the transmission of ascending and descending sensorimotor information between the brain and spinal cord. These findings provide support for the notion that the insult to the developing brain results in widespread structural changes within both the brain and spinal cord that subsequently results in adverse effects on motor outputs and the sensorimotor system more broadly.

The brainstem is a critical structure involved in mobility and locomotion that has been largely understudied in persons with CP. Lying at the base of the brain, it connects the cerebrum to the spinal cord. Comprised of the midbrain, pons, medulla, superior cerebellar peduncle (SCP), and other structures, the brainstem is largely responsible for many autonomic processes, including regulation of the cardiovascular and respiratory systems. However, it is also crucially involved in processes relating to locomotion and sensorimotor integration. The brainstem provides a conduit for the corticospinal tracts to send motor commands to the spinal cord, as well as for the dorsal medial lemniscus and spinothalamic tracts to transmit incoming somatosensory and pain information from the spinal cord to the cortex. Thus, structural changes within the brainstem may affect the integrity of both incoming and outgoing sensorimotor information along these tracts. Additionally, several other tracts originate within various nuclei in the brainstem that play a critical role in mobility. The rubrospinal tract originates from the red nucleus in the midbrain, which is a descending motor tract highly involved in sensorimotor integration within the spinal cord, the control of posture and gait, and skilled movement control of the forelimb digits [20–22]. Another tract in this area, the reticulospinal tract, originates in the reticular formation, where it regulates locomotion, balance, postural control, and muscle tone [23–27]. Thus, alterations in the volume within the brainstem may reflect changes to the cell size or density of synaptic connections within these different nuclei and subsequently have an adverse effect on mobility.

One previous study has identified that the total brainstem volume is decreased in persons with CP [28]. However, the specific areas within the brainstem that showed altered structure were not investigated, and it remains unknown whether structural abnormalities within the brainstem could potentially play a role in the uncharacteristic mobility seen in CP. The objective of this study was to use MRI and more advanced computational neuroanatomical approaches to investigate the potential structural alterations within the sub-structures of the brainstem in persons with CP and determine whether these changes were connected with the extent of the mobility impairments. First, we hypothesized that participants with CP would exhibit alterations in brainstem structures when compared with their neurotypical (NT) peers. Specifically, we hypothesized that there would be alterations in the volumes of the midbrain, pons, medulla, and superior cerebellar peduncle (SCP). Secondarily, we hypothesized that these structural alterations would be connected with the extent of the gait impairments seen in this patient population.

## Methods

### Subjects

The Institutional Review Board reviewed and approved the protocol for this investigation. This experimental work conformed to the general standards set by the Declaration of Helsinki. Participants and/or their guardians provided written informed consent, and all participants assented to participate in the investigation. Structural imaging of the brainstem was acquired from 64 participants. Twenty-six of the participants had a diagnosis of CP (Age = 16.3 ± 1.0 yrs, Females = 12, GMFCS levels I-IV) while thirty-eight served as NT controls (Age = 14.3 ± 0.4 yrs, Females = 14). The controls had no known neurological or musculoskeletal impairments. Furthermore, the participants with CP were excluded from this investigation if they had an orthopedic surgery or anti-spasticity treatments within the previous six months.

### Gait Analysis

Subjects walked at their fast-as-possible walking speeds across a mat that registered their digital footprints (GaitRITE, Sparta, NJ). A fast-as-possible walking speed was utilized to better expose the walking capacity of the participants. The data collected from the mat was used to calculate the subject’s walking velocity, step length, and step width. Each subject completed two fast-as-possible walking speed trials. The average across these trials was used as the outcome variable. The participants with CP were allowed to use any assistive device for the walking test (i.e., forearm crutches, walking, ankle-foot-orthosis, etc.).

### Structural MRI Volumetric Analysis

**Figure 1:**
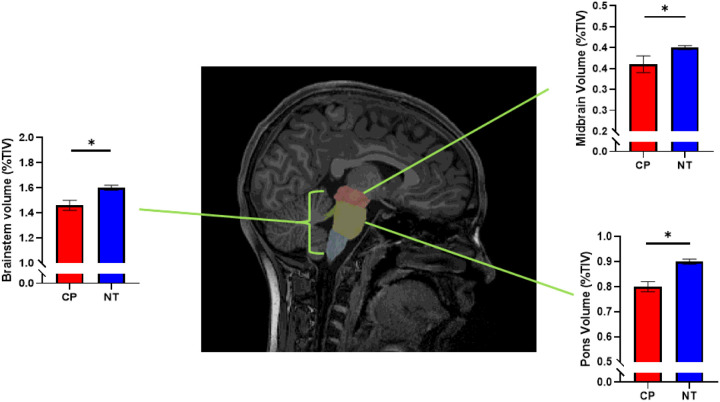
Example of the segmented brainstem in a representative participant with CP showing the midbrain (red), pons (dark yellow), medulla (blue), and superior cerebellar peduncle (SCP; green). Bar graphs depict differences in volume among the segmented areas of the brainstem between persons with CP and NT controls. The persons with CP had reduced brainstem volume, with specific reductions within the midbrain and pons (*p*s < 0.05).

All participants underwent an MRI scan on a 3-Tesla Siemens Prisma MRI scanner using a 32-channel head coil. High resolution T1-weighted images were collected using an MPRAGE sequence (192 slices, TR = 2400 ms, TE = 2.05 ms, FOV = 256 mm, flip angle = 8°, voxel size = 1 × 1×1 mm). We subsequently performed volumetric processing and segmentation using the FreeSurfer software package (http://surfer.nmr.mgh.harvard.edu). We additionally used the FreeSurfer brainstem pipeline to process our T1 images [29]. This pipeline has previously been shown to reliably segment the brainstem relative to other methods [30]. Briefly, the brainstem was segmented into the following subareas (midbrain, pons, medulla oblongata, and SCP), and volumes were extracted for each area (Fig. 1). The brainstem volumes were then normalized to the total intracranial volume (TIV) of each participant. These segmented brainstem subfields were manually verified per participant for errors. No manual interventions were performed on the data. For a full description of this pipeline, see Iglesias et al., 2015.

### Statistical Analysis

Independent samples t-tests were used to compare volumes of the whole brainstem, midbrain, pons, medulla, SCP, and gait parameters between the persons with CP and NT controls. Correlations between brainstem volumes and gait parameters were then evaluated using Pearson’s Correlations. All statistical comparisons were performed using JASP (0.16.2.0).

## Results

### Spatiotemporal Kinematic Analysis

Consistent with the literature, the participants with CP had a slower fast-as-possible walking velocity (CP = 1.40 ± 0.11 m/s, NT = 1.96 ± 0.04 m/s, *P* < 0.001), a smaller step length (CP = 0.65 ± 0.03 m, NT = 0.85 ± 0.02 m, *P* < 0.001), as well as larger step widths (CP = 0.13 ± 0.01 m, NT = 0.09 ± 0.01 m, *P* < 0.001) when compared with their NT peers. Overall, these results support the impression that persons with CP have an atypical gait.

### Structural MRI Volumetric Analysis

TIV was smaller in the persons with CP in comparison to the NT controls (CP = 1.45 × 10^6^ ± 3.38 × 10^2^ mm^3^, NT = 1.58 × 10^6^ ± 2.83 × 10^2^ mm^3^, *P* = 0.004). After correcting for TIV, the persons with CP had significantly smaller brainstem volumes compared with the NT controls (CP = 1.46 ± 0.04%, NT = 1.60 ± 0.02%, *P* = 0.005). The volume of the midbrain was also smaller in persons with CP (CP = 0.36 ± 0.02%, NT = 0.40 ± 0.00%, *P* = 0.026), and the volume of the pons was significantly smaller in the persons with CP in comparison to the NT controls (CP = 0.80 ± 0.02%, NT = 0.90 ± 0.01%, *P* < 0.001). The volumes were similar between groups within the medulla (CP = 0.27 ± 0.01%, NT = 0.28 ± 0.01%, *P* = 0.150) and the SCP (CP = 0.02 ± 0.00%, NT = 0.02 ± 0.00%, *P* = 0.200). Overall, these findings imply that there are prominent structural alterations within the brainstem in persons with CP.

### Correlations

**Figure 2:**
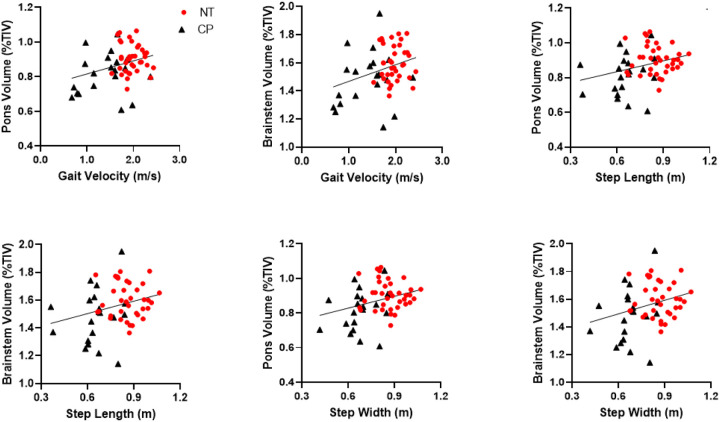
Scatterplots depicting the relationships between gait variables and brainstem sub-volumes. The scatter plots reveal that a faster walking velocity, longer step length, and wider step width were associated with a larger brainstem and pons.

Next, we determined whether the decreases in volume within the brainstem, midbrain, and pons in persons with CP were associated with poorer gait performance. Across all participants, a faster walking velocity was associated with a larger brainstem (r = 0.31, *P* = 0.018) and a larger pons (r = 0.31, *P* = 0.019). (Fig. 2). Increased step length was also associated with a larger brainstem (r = 27, *P* = 0.040) and a larger pons (r = 0.30, *P* = 0.025) (Fig. 2). Finally, a larger step width was associated with a larger brainstem (r = 0.28, *P* = 0.034) and larger pons (r = 0.30, *P* = 0.025) (Fig. 2). The midbrain volume was not associated with gait velocity, step length, or step width (p’s > 0.05). Altogether, these correlations implied that those with a larger brainstem volume and pons tended to have better gait performance.

## Discussion

In the current study, we found that persons with CP had smaller brainstems than their NT peers, including specific volume reductions within the pons and midbrain. Furthermore, our novel results demonstrated that the reduced brainstem volumes and pons volume were associated with reduced gait velocity, step length, and step width. These novel results support the notion that the early insult to the developing brain in persons with CP results in structural changes at the level of the brainstem that ultimately contribute to the mobility impairments that are ubiquitously observed in this population. Further discussion of these novel experimental findings appears in the following sections.

Our structural MRI analysis revealed that the entire brainstem, the pons, and the midbrain had reduced volumes in the persons with CP in comparison to the NT controls. These results expand on the current literature, which has illustrated that the insult to the developing brain in persons with CP has widespread effects on structural integrity throughout the CNS, including many areas of the neocortex, upper spinal cord, and thalamocortical tracts [12, 15, 17–19, 31]. Importantly, the brainstem is comprised of both long axons of efferent and afferent tracts, as well as various nuclei [29]. Thus, reductions in volume could be indicative of either reduced gray matter or white matter that ultimately impacts the many different functions in which the brainstem is involved, including vision, hearing, motor control, and sleep regulation in the midbrain, to respiration, cardiac function, and reflex control in the pons and medulla. Alterations in gray matter may indicate a loss of cell bodies, decreased cell number, or reduced synaptic density. Reductions in the white matter may be indicative of reduced myelination that could affect the integrity of signals being propagated between the brain and spinal cord. In either case, each of these detriments to the brainstem’s structure may have adverse effects on sensorimotor functioning and mobility.

Corroborating this idea, we found that further decreases in volume within the brainstem and pons were significantly associated with slower walking velocity, smaller step lengths, and smaller step widths. Importantly, the brainstem provides a conduit for the sensorimotor tracts that connect the cortex and cerebellum to the spinal cord, in which the efferent corticospinal and afferent dorsal medial lemniscus pathways pass through. The corticospinal tracts are involved in the facilitation of lumbar spinal motoneurons and interneurons that are integral for lower extremity muscle activation during voluntary contraction and skilled locomotion [32, 33]. Presumably, damage to the integrity of the corticospinal or sensory tracts at the level of the brainstem could result in difficulties with processing incoming somatosensation, sending motor commands, and sensorimotor integration. Alternatively, reductions in brainstem volume could also reflect structural changes to the gray matter of the various nuclei that serve as the origins of other prominent efferent tracts. For example, the rubrospinal and reticulospinal tracts originate within the red nucleus and reticular formation, respectively. These tracts are intricately involved in controlling the velocity and direction of movement, postural control, locomotion, and sensorimotor integration [20–22, 34]. As such, alterations within these nuclei could disrupt these processes and result in the deficits in gait velocity, step width, and step length that were found in the persons with CP.

The emerging clinical question that arises from these findings is whether improvements in mobility may result in structural changes that can be identified at the level of the brainstem. Numerous studies have demonstrated that sensorimotor and mobility improvements following therapeutic intervention are associated with neuroplastic changes within the somatosensory cortices [35, 36], indicating that the early maladaptive changes within the CNS may have the potential to be reversed. Whether this is the case within the brainstem is unknown, but such data is critical as it would provide novel insight into its candidacy as a therapeutic target. For example, deep brain stimulation has been performed on the brainstem in patients with Parkinson’s Disease, with research suggesting it can have beneficial effects on motor outcomes (51). Further, brainstem function may also impact persons with CP in areas outside of the sensorimotor system. For example, the brainstem is highly involved in pain processing [37], and the altered volumes reported here could contribute to the increased pain perception reported by many individuals with CP [38–41]. The brainstem is also highly involved in respiration, and changes to its structural integrity may relate to the increases in energy expenditure prominent within this population [27–30]. These areas and others should figure prominently into future work to shed light on the impact of brainstem dysfunction in CP.

### Limitations

Before closing, it is worthwhile to mention study limitations. First, this study focused solely on the brainstem, and we did not look at differences in volume across the cortex. Second, there were no measures of motor functioning included within this study, and it may be useful to determine how these structural alterations within the brainstem are associated with changes in upper and lower extremity motor functioning. Finally, there were no relevant therapeutic interventions within this study, and such information would help to decipher whether structural alterations within the brainstem are able to be changed alongside the improvements in clinical motor functioning that are seen following an effective therapeutic intervention.

## Conclusion

In this study, we determined that persons with CP have decreased brainstem volume, driven by reduced volume in the pons and midbrain. Further, we identified that these decreased volumes were correlated with decreased gait velocity, reduced step length, and smaller step width. Taken together, these results suggest that structural alterations in the brainstem may contribute to the mobility deficits commonly observed in persons with CP. These findings pave the way for future work to explore the other impacts that altered structure within the brainstem may have in CP, as well as its overall potential as a therapeutic target.

## Data Availability

Data will be made available upon reasonable request to the corresponding author, Max Kurz.

## Key Points

- Total brainstem, pons, and medulla volumes are decreased in people with cerebral palsy (CP).
- The reduced brainstem volumes are associated with reduced gait velocity and step length.
- Structural changes within the brainstem may contribute to clinical mobility impairments in people with CP.
